# Community based saving groups: an innovative approach to overcome the financial and social barriers in health care seeking by the women in the rural remote communities of Pakistan

**DOI:** 10.1186/s13690-017-0227-3

**Published:** 2017-08-10

**Authors:** Babar Tasneem Shaikh, Qayyum Noorani, Shazia Abbas

**Affiliations:** Aga Khan Foundation, Level 9, Serena Business Complex, Islamabad, Pakistan

**Keywords:** Community based savings groups, Community midwives, Maternal, Health care seeking, Newborn and child health

## Abstract

**Background:**

In remote rural areas of Pakistan, access to the maternal, newborn and child health (MNCH) care provided by a skilled health provider is quite difficult. There are many reasons such as women’s restricted social mobility, lack of education, disenfranchised in decision making and poverty. To overcome these barriers and impediments in district Chitral, which is the largest territory in terms of geography in province Khyber Pakhtunkhwa, local women of reproductive age, were mobilized to form the Community Based Saving Groups (CBSGs) at the village level. In these CBSGs, they pool-in their money, and then provide soft loans to the expecting mothers to meet the expenses of delivery. Simultaneously, young literate women were identified from the local communities; they were trained as Community Midwives (CMWs), using national MNCH curriculum, and later deployed in their respective villages within the district. This study captured their perceptions about the formation of CBSGs to overcome the financial and social barriers, and subsequent use of CMW services.

**Methods:**

A qualitative enquiry was conducted with the delivered mothers and their husbands through gender specific separate focus group discussions, with CBSG members and with non-members in four different sites of District Chitral.

**Results:**

CBSG member women were far more aware on health issues. Information sought from these forums brought a noticeable change in the health seeking practices. Seeking care from a trained birth attendant in the community became easier. Women associated with the CBSGs as members, expressed an increased access to money for utilizing the CMW services, better awareness on MNCH issues, and empowerment to decide for seeking care. CBSG have been an instrumental platform for social networking, helping each other in other household matters.

**Conclusion:**

Women have started using the services of CMW and the CBSGs have actually helped them overcome the financial barriers in health care seeking. Moreover, the CBSGs became a medium to improve the awareness of service availability, understanding the MNCH issues, and timely utilization of MNCH services.

## Background

Like many other rural remote communities in Pakistan, district Chitral in the north-west of Khyber-Pakhtunkhwa province in Pakistan presents the most difficult terrain, harsh cold weather, poverty, conservative culture, and limited facilities of education and healthcare. The district shares its border with Gilgit-Baltistan to the east, with Swat and Dir to the south, and with Afghanistan’s Kunar, Badakshan and Nuristan provinces to the north and west. A narrow strip of Wakhan corridor separates Chitral from Tajikistan in the north. Chitral is connected to the rest of Pakistan by two road routes, the Lowari Pass from Dir and the Shandur Top. Both routes are closed in winter [1]. As a result, the access to health care and especially the skilled birth attendants (SBAs), poses a significant challenge for the expectant mothers. The district presents high levels of maternal and newborn morbidity and mortality as compared to other neighboring district of the province [2]. Global as well as local experience shows that delays in seeking care, reaching care, and receiving care are related to inadequate knowledge, logistical and financial constraints, gender insensitive health policies and programs, gaps in the health service provision and coverage, and low levels of family and community support [3, 4].

In district Chitral, lack of public transport, too high fares, distantly located health facilities, travel time, and uncertain availability of the trained healthcare provider pose serious challenges in seeking appropriate health care in time [5]. Amidst a majority of population subject to abject household poverty (around US$2 /day), most of the deliveries (64%) occur at homes, without the assistance of a skilled birth attendant [6]. Historically, female illiteracy has also contributed to women’s inability to effectively understand and act on their health needs [7, 8]. Almost half of the women (46%) had never attended a school and two third were illiterate, in communities typically ascribing to conservative socio-religious dictates on women’s social mobility, even for emergency health care seeking [9].

### Program intervention

Poverty and lack of finances for travel have restrained most of the women in seeking health care from a skilled birth attendant. As a result, most of the deliveries occur at home, with the aid of the village midwife (*dai*) or a senior woman relative or a neighbour [5]. This unsafe practice and behaviour has had serious implications on the health of the mother and the newborn in the area. Four years project duration [2012–2015] aimed at overcoming the financial barriers of the pregnant women seeking care from the skilled birth attendants (SBAs) in district Chitral; and ensuring the availability of a trained birth attendant i.e. community midwife in each intervention village of the district.

Local women particularly of the reproductive age were sensitized and mobilized to form at least one Community-Based Savings Groups (CBSGs) in each village. More than 400 such groups were formed across the district. The membership of the group was voluntary and with no fix joining fee or deposit; it could be as low as PkRs10 only. Each CBSG comprised 10 to 30 women who deposited and pooled their savings (per their capacity and affordability). Women were given shares against their money deposited (each share costs PkRs10) and a record of individual’s savings was maintained in a register. Then on receiving a loan request, the group was convened to decide on lending the money internally through a consensus at a pre-defined, mutually agreed upon interest rate. The locked money box, the key and the record book were kept with three different members to ensure secrecy and transparency. Loan is paid back on a mutually agreed time frame and on a soft interest rate, and late return charges or the fate of defaulter cases were also decided among the group with consensus [10]. Moreover, CBSGs became a conducive platform for the village women to participate, interact, and discuss about their reproductive health issues, exchange advantages of skilled delivery, and highlight the role of CMWs. The forum largely contributed to the women’s financial autonomy and empowerment. The CBSG model was developed based on the global experience to provide a simple, transparent, cost-effective entry level financial services for poor rural populations with fundamental promise that funds saved now will be available in the future, and also allowing a space for community interaction [11].

For deploying skilled birth attendants, partnership memorandum was signed with the National Maternal, Newborn & Child Health (MNCH) Program. Twenty eight young women identified with the help of lady health workers and the village elders; were recruited from the remote locations. They were imparted a training of 18 months as community midwives (CMW), as per the standard national curriculum [12]. In Pakistan’s rural communities, CMWs are the only accessible, clinically trained provider for the continuum of care: antenatal and post-natal care, birth preparedness and complication readiness counselling, skilled delivery, and postpartum family planning [13]. Considering the bit of hesitation associated with the acceptance of the role of young CMWs in the communities, community support system was developed. One CMW was deployed in one village, but covering 2–3 more adjacent villages. For each CMW, one Village Health Committees (VHCs) was established and linked with a cluster of 8–10 CBSGs.

### Study objectives

This qualitative descriptive study was part of the larger study which attempted to answer the question i.e. whether membership in CBSGs contributes to increased awareness of service availability, understanding of MNCH issues, in addition to greater utilization of MNCH services in the community, specifically those offered by CMWs [14]. Hence this qualitative descriptive part endeavoured to document the role of the CBSGs in overcoming the ‘financial barriers’ and subsequent utilization of the services of a skilled birth attendant i.e. CMW. It also assessed whether CBSGs have actually been instrumental in overcoming some of the ‘social barriers’ to access skilled health providers, particularly the CMWs.

## Methods

### Data collection tool

After a thorough review of the literature, we developed a question guide comprising probes to record the contextual insights about the role and benefits of CBSG membership, especially with regard to health awareness, health seeking practices, their experience in the last delivery and the costs incurred, and use of MNCH services offered by the newly deployed CMWs. The study also looked into other social effects produced by community savings, financial autonomy, decision-making authority, degree of empowerment, and group solidarity. Men were asked to comment on their role in the whole cycle of pregnancy and child birth; merits and de-merits of CBSGs, and what if a similar CBSG is created for them also. The question guide was pilot tested in one of the villages with one group of women to see the composition, sequence, language, duration and level of understanding of the different probes to be asked. Minor adjustments were made for the language and sequence of probes. The FGD guide was eventually translated into a local language *‘Khowar’*.

### Data collection

We conducted Focus Group Discussions (FGDs) in order to explore a series of inter-related questions and issues. Of the 28 locations, where CBSGs were formed and the CMWs were deployed and working, four locations were identified for conducting the research in order to capture the diversity in geography, ethnicity and the socio economic status within the program area. With this criterion, two locations were randomly selected from each of the two tehsils of the study district. FGD participants were asked to share the barriers health care seeking especially the financial issues, benefits of CBSGs in terms of access to money, utilization of CMW service, and improved awareness on MNCH issues. Separate FGDs were conducted with women who have still not opted to become a member of the CBSG, and their husbands to record the reasons for not joining. The study conducted 16 FGDs to reach the data saturation [4 each with women member CBSGs and their husbands; and 4 each with women non-members and their husband]. Participants for the FGDs at each of the four locations were:

1. women who had delivered within the last quarter and were members of a CBSG;
2. husbands of women CBSG member who had delivered within the last quarter;
3. women who had delivered within the last quarter and were not members of a CBSG; and
4. husbands of women who had delivered within the last quarter and were not a CBSG member.

Each FGD comprised 6 to10 men or women. The meetings were held at a neutral place (schools, community centres etc.) in the village, agreeable and accessible to all the invited participants. The duration of one discussion spanned over an hour on average. To identify participants, the data collectors visited prospective participants’ homes and if women and men agreed, informed consent was obtained and invitation to FGD was extended. During the FGDs, participation diagrams were made so as to ensure that an equal opportunity was provided to all the FGD participants to speak and contribute to the discussion. Verbatim notes of the FGDs were transcribed from the local language ‘Khowar’ to Urdu to provide a record of what was said.

### Data analysis

Qualitative data, in turn, was analysed manually, according to the principles of grounded theory, which can be defined as the generation of theories about a social phenomenon, and to develop higher level understandings that are ‘grounded’ in, or derived from the systematic analysis of data. Such an approach is most appropriate when the study of social interactions or experiences aims to explain a process, and not to test or verify an existing theory [15]. Hence, in this study, the social phenomenon studied was *‘the extent to which CBSG membership facilitated women’s use of CMWs’ services’*.

Data analysis was done manually, identifying nodes and sub-nodes to generate and classify the themes arising from the FGD transcripts. All the data relevant to each node was then examined, using a process called ‘constant comparison’. In this way, each item or aspect of the primary data was checked and compared to the larger data to establish the analytical categories. Later, the data was aggregated and analysed to develop the themes. Theoretical coding, memo writing and data-sorting were employed in the process of writing the first version of the manuscript. The codes and themes produced because of the preliminary analysis were then re-written and re-organised.

## Results

Participants of the FGDs were quite homogenous in socio-demographic characteristics. Most of the women who participated in the FGDs were housewives and poor, with an average household monthly income of less than PKR 10,000 (approx. US$100). Majority of the participants of FGDs were aware of a CMW working in their area, and most of them had already sought assistance from the CMWs during the last pregnancy. The CMWs had regular interaction with women during pregnancy, counseling, referral to next level facility and in providing post natal care. More than two-third women were associated with CBSGs either directly or through immediate family members. The qualitative results are compiled under the themes which emerged from the analysis of sub-nodes and nodes.

### Improving health awareness and practices

The participants admitted that they are better aware on the health issues than before. CBSG forums provided enabling environment for the counseling and health awareness sessions by CMWs and the supervisors. CBSGs became the forums for behaviour change communication on pregnancy, childbirth, breastfeeding, family planning and immunization. The findings of the FGDs revealed that health-seeking practices were changing among women who joined CBSGs a year ago.

*“We are more aware on issues regarding our own health and the health of the newborn. We try to follow what CMW tell us about our diet, child’s feeding, spacing between children* etc”. *(Women member CBSG).*

Study participants confirmed that due to regular interaction with CMW through group meetings, they developed the confidence on the ability and role of CMW to provide quality services during pregnancy. On the other hand, many male participants (husbands of the CBSG members) acknowledged that the CBSG’s membership enabled their women to access the counseling and health awareness sessions conducted by the CMWs. They reported that now women are actively seeking the support of their family for birth preparedness.

*“It is because of CBSG that I have been able to persuade my wife that CMW is based in our community; so it is better to avail her services”. (Husband-member CBSG).*

CBSG membership has not only empowered the women to take their decisions regarding the place of delivery; it has also helped to enhance members’ knowledge on importance of good diet, immunization and rest during the pregnancy.

### Utilization of CMW services

CBSG members were asked to discuss the role of CBSGs in facilitating the utilization of CMWs’ services.

*“Many a times, men are not at home; therefore I can take loan from CBSG in the time of need. Also I don’t want to take loans from relatives or neighbours”. (Women member CBSG).*

Husbands of women members appreciated the facilitating role of CBSG as:

*“I sold my motorcycle at my wife’s first delivery; second time I borrowed money from a village elder. This time, we have our own savings in CBSG, and with that money we can access the CMW”. (Husband-member CBSG).*

It was shared that CBSGs have been instrumental in establishing trust and social acceptability of the CMW. Community members were appreciative of the fact that CBSGs have become a forum to inform the community women about CMW’s skill and knowledge and that she had been deployed in their area to work as a trained birth attendant.

*“My wife has joined CBSG because it helps in understanding the issues of seeking care for ante-natal, delivery and post natal times, and now she has full confidence on the CMW for handling her delivery”. (Husband-member CBSG).*

Many participants confirmed that women are now consulting the CMWs for ante-natal check-ups and that only complicated cases are referred to the nearest hospital for appropriate treatment.

*“CBSG has introduced us to the CMW. We are now consulting her for regular check-up during the pregnancy. She has also informed us that in case of any complication, she can refer us to the nearest hospital”. (Women member CBSG).*

*“CBSG is a good mechanism for saving. We are consolidating our own money for needy times, particularly for emergency at the time of delivery of my wife”. (Husband-member CBSG).*

The newly deployed CMWs faced many challenges (lack of family permission, deployment outside their native village, late working hours etc.), and as a result few of them left the job. For improving their retention and performance, CBSGs played a pivotal role of providing the community support and recognition of their services.

*“CBSG conducts regular meetings with community women to promote CMW services. Many women still do not know about her”. (Women member CBSG).*

### Social benefits of CBSGs

Besides financial empowering of the women groups, and equipping them with appropriate knowledge on safe motherhood, the CBSG have been an instrumental platform for social networking, helping each other in other household matters such as for marriages and funerals, purchasing food items, establishing small businesses, or paying school fees for their children.

*“CBSG provides a platform for social networking, community support and exchanging our view on mother and child health. It is a good pass time too. We chat, we laugh and share our worries with each other”. (Women member CBSG).*

CBSG membership has helped to establish a greater degree of solidarity among the community members.

*“As compared to women who stay at home all day, we are more knowledgeable, smart and aware about social dynamics, and know how to utilize CBSG money to alleviate our poverty from our community, cover children’s school fees and other household related expenses (Woman member CBSG)”* *.*

Simultaneously, the older women members (mothers-in-law) shared that they used to insist on seeking delivery assistance from a local *Dai* (traditional birth attendant); but now they support their daughters in law to consult the CMW. In a way, CBSG has sensitized the elder generation too on the importance of safe delivery in the presence of a trained birth attendant.

By virtue of the CBSG membership, women gained knowledge and confidence to have their say in decisions pertaining to health and other matters in the family.

*“CBSG has increased in women’s confidence and empowered them in decision making for their own health”* *. (Husband-member CBSG).*

Some of the women started small businesses in partnership with other CBSG member women. Participants during FGD mentioned that CBSG has become an alternative banking system in their village. In addition, FGD participant shared that CBSGs provided a platform to extent and obtain community support and also to interact with each other, and share happiness and worries.

*“CBSG has provided our women an opportunity to socialize, to empathize and to be confidante of each other. They now know what is happening in the world. Moreover, women have become economically empowered”. (Women member CBSG).*

### Perceptions of women non-members and their husbands about CBSGs

From the discussions with women who have not yet become the member of any CBSG and their husband, it was noted that lack of information about CBSGs’ structure, its purpose, and membership requirements is keeping many people in oblivion about the advantages of becoming a member.

*“We are not well informed about this women saving group, and we are not sure of its real benefits. I am not sure whether my husband would allow me to go to a CBSG”. (Woman, non-member).*

Women, who are not yet the members, stated that they were hesitant to ask their husbands for the money, initially required to join a CBSG. Some men and women perceived that their financial situation does not permit them to join CBSG.

*“We might not have extra money every now and then to contribute to the group; and I don’t want to burden my husband by asking him money for such savings”. (Woman, non-member).*

Some people also had a negative prior experience with similar groups, and/or organisations, which never provided them any substantive benefit.

*“Past experience with such associations, groups and organizations is not pleasant. There has been too much wastage of time, especially in discussing matters other than health”. (Woman, non-member).*

In separate FGDs with men whose wives are still not members of CBSG, were asked to share their ideas. Many of them stated that national identity card (NIC) is a mandatory requirement to join a CBSG, and that many women do not possess one in this remote area. However, after understanding the details about CBSG, many of them agreed to get the NICs for their wives and to allow their women to join the CBSG.

*“Having listened to the advantages of CBSG, we can now confidently recommend our women and ask other men to send their women for membership in CBSG” (Husband, non-member).*

Some of the men were concerned about the odd distances that women have to cover on foot to reach the CBSG site, and that restrained them to let the women join the group.

*“Since we do not have a CBSG in our own village, our women will have to travel to adjacent village which is time consuming, and it is also not secure to send them alone”. (Husband, non-member).*

All of them offered their unanimous support and agreement to establish more CBSGs in the rest of the villages. Men stated that by having an alternative money saving system in their community, they would also be able to develop solutions to a number of other issues such as borrowing loans for small businesses, paying children school fee, buying household items, and bearing expenses of their daughters’ marriages.

## Discussion

Our principal findings suggest that women can be enabled to seek appropriate and skilled health care if they do not have a financial constraint; and that a local trained health provider is far more accessible and acceptable as compare to somebody far located and not having the cultural insights of the community. These findings resonate the fact that financial situation of a family govern the health care seeking among the expecting mothers [16, 17]. Other studies and literature from the region and Pakistan in general confirm the fact that health-seeking behaviors are also closely associated with socioeconomic determinants in any community [4]. Due to financial constraints, most of the women have preferred home-based delivery care [18, 19]. Our study confirms that in a developing country’s health system, where there are a few doctors and other SBAs, CMWs are the easily accessible and would be the preferred cadre of trained workers as compared to the traditional birth attendant to fill in the gap, and could be very much acceptable to the local community. Another finding of our study is that due to regular interaction of the CMWs with women through the CBSG forum, CMWs gain acceptance in the community, eventually preventing complications of pregnancy and childbirth, with an early and timely diagnosis of any danger signs and prompt referral to the hospital. It is a well-documented fact that such participatory intervention helps women living in remote rural areas with limited resource to develop appropriate health seeking practices of utilizing the services of trained birth attendants, thereby showing improvements in birth outcomes in a disadvantaged population. [20, 21] This remote under-served geography of the north-west province presents more or less similar picture of more than 65% of Pakistan population living in the poverty struck rural areas, facing challenge of accessibility to quality healthcare [22].

Such women centred group meetings are the best forum to convey important health awareness messages about MNCH issues, birth preparedness and importance of taking well-informed and timely decisions for seeking health care [23, 24]. Our study furthered the concept in the context of Pakistan, where modest savings by the CBSG members helped them overcoming the financial constraints, and hence enabled the extremely poor women to decide about the travel to reach out and avail the services of CMWs. This is suggestive of the women’s group action learning and experience sharing need to be continued for knowledge improvement and behaviour change in birth practices, saving newborn lives, and nutrition [4, 21, 25, 26] .

This was the first research, looking at such phenomenon in this kind of remote setting. We not only looked at the women’s believes, perceptions and practices, but also involved men to record their views on the subject. Results of this research may not be extrapolated for the entire country, because of the wide cultural diversity across the country. Perspectives of CMWs could be another interesting facet to look at, after passage of some time. Although women did not mention any specific short comings of the CBSGs; yet it would be interesting to go back and ask the same after the lapse of some time. A mix method and multiple sites research would be even more beneficial for generating an empirical evidence to suggest the government, and health department in particular, for scaling up of such initiatives. Nevertheless, the national and provincial MNCH programmes may consider scaling up this experience in other similar settings of the country.

## Conclusions

The CBSGs provided an enabling and women-empowering platform to influence health seeking practices around birth preparation and the choice of health provider. This community-based approach helped in improving the awareness of service availability, understanding the MNCH issues, utilization of MNCH services, and in overcoming the social and financial constraints, which the women of Pakistan face while seeking health care from a skilled provider.

## References

[1] Chitral. https://en.wikipedia.org/wiki/Chitral [accessed 30 Sept 2016].

[2] National Institute of Population Studies & Macro International. Pakistan Demographic and Health Survey 2006–07. Islamabad: National Institute of Population Studies & Macro International; 2008.

[3] World Health Organization. Beyond the numbers: Reviewing maternal deaths and complications to make pregnancy safer. Geneva: WHO; 2004. 10.1093/bmb/ldg009 14711752

[4] Shaikh BT, Haran D, Hatcher J (2008). Women’s social position and health seeking behaviours: is healthcare system accessible and responsive in Pakistan?. Healthcare Women Int.

[5] Shaikh BT, Khan S, Maab A, Amjad S (2014). Emerging role of traditional birth attendants in mountainous terrain of Chitral District, Pakistan. BMJ Open.

[6] National Institute of Population Studies & Macro International. Pakistan Demographic and Health Survey 2012–13*.* Islamabad: 2013.

[7] Pittman PM (1999). Gendered experiences of health care. Int J Qual Health Care.

[8] Winkvist A, Akhtar HZ (2000). God should give daughters to rich families only: attitudes towards childbearing among low-income women in Punjab, Pakistan. Soc Sci Med.

[9] Pakistan Poverty Alleviation Fund. Situation Analysis & Baseline Surveys for poverty reduction through rural development in KPK, FATA & Baluchistan: District Profile Chitral. Islamabad: 2014.

[10] Noorani Q, Azam I, Shaikh BT, Abbas S, Ranasinghe T (2013). Role of community based savings groups (CBSGs) in enabling greater utilization of community midwives in Chitral District of Pakistan.

[11] Ashe J (2009). Savings-led microfinance and saving for change.

[12] Research & Development Solutions. The community midwives programme in Pakistan. Policy briefs series no 20. Islamabad: Research & Development Solutions; 2012.

[13] Mumtaz Z, Brien OB, Bhatti A, Jhangri GS (2012). Are community midwives addressing the inequities in access to skilled birth attendance in Punjab, Pakistan? Gender, class and social exclusion. BMC Health Serv Res.

[14] Noorani QA, Azam SI, Shaikh BT, Ranasinghe T, Abbas S, Wali S, Rippey P, Javed W (2013). Role of community based savings groups (CBSGs) in enabling greater utilization of community midwives in Chitral District of Pakistan. BMC Preg Childbirth.

[15] Green J, Pereyaslov D, Ahmedov M, Balabonova D (2010). The use of qualitative methodologies in health services/systems research in low and middle income settings: a narrative literature review.

[16] Agus Y, Horiuchi S, Porter S (2012). Rural Indonesian women’s traditional beliefs about antenatal care. BMC Res Notes.

[17] Hassan H, Jokhio AH, Winter H, MacArthur C (2012). Safe delivery and newborn care practices in Sindh, Pakistan: a community-based investigation of mothers and health workers. Midwifery.

[18] Barayal YR, Lyons K, Skinner J, van Teijlingen ER (2010). Determinants of skilled birth attendants for delivery in Nepal. Kathmandu Uni Med J.

[19] Titaley CR, Hunter CL, Dibley MJ, Heywood P (2010). Why do some women still prefer traditional birth attendants and home delivery? A qualitative study on delivery care services in west Java Province, Indonesia. BMC Preg Childbirth.

[20] Manandhar DS, Osrin D, Shrestha BP, Mesko N, Morrison J, Tumbahangphe KM, Costello A (2004). Effect of a participatory intervention with women's groups on birth outcomes in Nepal: a cluster-randomised controlled trial. Lancet.

[21] More NS, Bapat U, Das S, Alcock G, Patil S, Porel M, Vaidya L, Osrin D (2012). Community mobilization in Mumbai slums to improve perinatal care and outcomes: a cluster randomized controlled trial. PLoS Med.

[22] Shaikh BT, Hatcher J (2005). Health seeking behavior and health services utilization in Pakistan: challenging the policy makers. J Public Health (Oxford).

[23] Azad K, Barnett S, Banerjee B, Shaha S, Khan K, Rego AR (2010). Effect of scaling up women's groups on birth outcomes in three rural districts in Bangladesh: a cluster-randomised controlled trial. Lancet.

[24] Kesanta J, Andre B. Impact of Women Empowered through Community Savings Groups on the Wellbeing of their Families: A Study from Mgubwe, Tanzania Interdisciplinary Journal of Best Practices in Global Development 2015. http://knowledge.e.southern.edu/cgi/viewcontent.cgi?article=1004&context=ijbpgd [Accessed 4 Jan 2017].

[25] Tinker A, Parker R, Lord D, Grear K (2010). Advancing newborn health: the saving newborn lives initiative. Glob Public Health.

[26] Waller MK (2014). Empowering women through savings groups: a study from the wellness and agriculture for life advancement (WALA) program.

